# Cumulative dairy cow genetic change from selection and crossbreeding over the last 2 decades in New Zealand closely aligns to model-based predictions published in 2000

**DOI:** 10.3168/jdsc.2020-0043

**Published:** 2021-01-22

**Authors:** N. Lopez-Villalobos, H.T. Blair, D.J. Garrick

**Affiliations:** AL Rae Centre for Genetics and Breeding, School of Agriculture, Massey University, Palmerston North 4442, New Zealand

## Abstract

A deterministic model was developed in 1998 to evaluate the concurrent effects of selection and crossbreeding on the rate of genetic gain and increases in productivity of New Zealand dairy cattle over the ensuing 25-yr period. The predictions of today's breed composition of the national dairy herd and genetic trends for body weight and lactation yields of milk, fat, and protein are compared with today's actual values. Selection was assumed to use an index that included live weight and lactation yields of milk, fat, and protein. Mating strategies involving the Holstein-Friesian (F), Jersey (J), and Ayrshire (A) breeds were evaluated. Effects of heterosis and age were included to calculate phenotypic live weight and yields of milk, fat, and protein per cow. At the time the model was developed, New Zealand had an across-breed evaluation system, but only straightbred bulls were used after progeny testing based on records of straightbred and crossbred daughters. The model predicted that if crossbred cows and bulls could be considered as bull parents, faster rates of genetic gain may result because of increased selection intensities in the cow to breed bull selection pathway. This scenario transpired, and the best bulls and cows for farm profit were used regardless of breed. Under that mating strategy for the 2018 birthyear, the model predicted the national breed composition would be 11% F, 34% J, 52% F×J, and 2% A; the actual breed composition was 36% F, 9% J, 53% F×J, and 1% A. The model-predicted annual genetic gains would be 16.7 L of milk, 1.2 kg of fat, 1.5 kg of protein, and −0.7 kg of body weight; the realized annual improvements were 13.6 L of milk, 1.31 kg of fat, 1.17 kg of protein, and −0.36 kg of body weight. Predicted long-term responses to selection can closely mirror realized improvements, confirming the value of modeling to inform animal breeding decision-making.

## Highlights

• Genetic trends for lactation yields of milk, fat, protein, and cow BW in New Zealand dairy cattle align to model-based predictions
• Actual proportion of Holstein-Friesian × Jersey crossbreds closely align to proportion predicted by the model
• Predicted long-term responses to selection can closely mirror realized improvements, confirming the value of modeling to inform animal breeding decision-making

## Graphical Abstract

**Figure:**
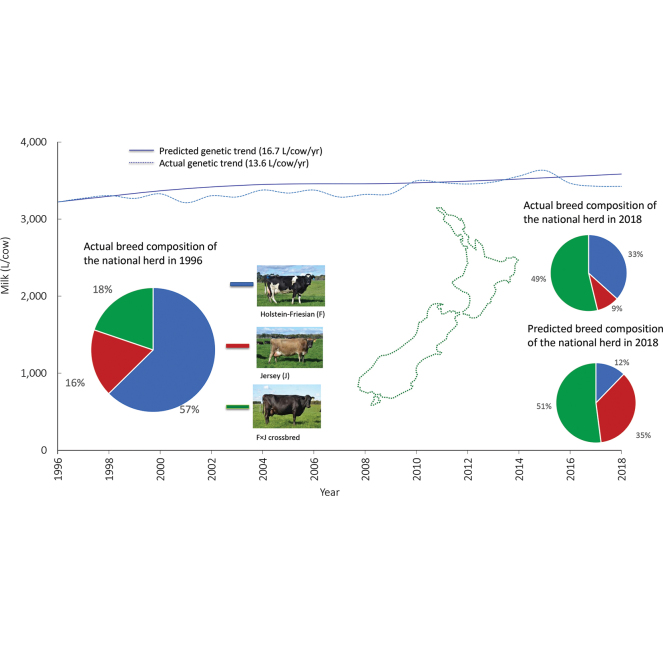
**Summary:** A deterministic model was developed in 1998 to evaluate the concurrent effects of selection and crossbreeding on the rate of genetic gain and increases in productivity of New Zealand dairy cattle over the subsequent 25-yr period. Despite many national and international changes in political, economic, and management circumstances, the predicted long-term responses to selection and crossbreeding options closely mirrored genetic trends for milk production and changes in the breed composition of the national herd. This outcome should give confidence to scientists, decision-makers, and funders that long-term predictions of the genetic impact of technological developments can be accurately modeled.

A deterministic model was developed in 1998 to evaluate the concurrent effects of selection and crossbreeding on the rate of genetic gain and increases in productivity of New Zealand dairy cattle over the subsequent 25-yr period. Despite many national and international changes in political, economic, and management circumstances, the predicted long-term responses to selection and crossbreeding options closely mirrored the genetic trends for milk production and changes in the breed composition of the national herd. This outcome should give confidence to scientists, decision-makers, and funders that long-term predictions of the genetic effect of technological developments can be accurately modeled.

Selection and crossbreeding are 2 strategies that can be used to increase farm profitability (Lopez-Villalobos et al., 2000a,b; Clasen et al., 2020). Models that simulate selection schemes for the genetic improvement of dairy cattle in combination with widespread use of crossbreeding strategies to exploit heterosis effects for production, fertility, and survival are scarce in the literature. Lopez-Villalobos et al. (2000a) developed a deterministic model to simulate the long-term effects of selection and crossbreeding on the annual rate of genetic gain and productivity of the New Zealand dairy industry over 25 yr. The objective of the current study was to compare today's breed composition of the national dairy herd and genetic trends for body weight and lactation yields of milk, fat, and protein predicted by the model with today's actual values. This comparison will validate whether long-term predictions of the genetic effect of technological developments can be accurately modeled.

The model developed 22 yr ago considered the numbers of cows and bulls and their base genetic merit that were representative of the 1996 to 1997 production season with age structures at equilibrium. The cow population was classified according to their breed composition and number of generations of artificial breeding male ancestors on the maternal side of their pedigree. Numbers of animals were updated each year using a herd-growth model (Upton, 1989).

The aggregate genotype (**T**) was deﬁned as T = Σv_j_G_j_, where v_j_ = economic value (in $) of trait j, and G_j_ was the additive genetic value of the selection candidate for trait j. Economic values were −NZ$0.427/kg of live weight, −NZ$0.05/L of milk, NZ$0.47/kg of fat, and NZ$4.054/kg of protein. An estimate of T was defined as breeding worth and was calculated as breeding worth = Σv_j_EBV_j_, where EBV_j_ is the estimated breeding value for trait j. Traits included in breeding worth were the same as those included in T. The economic values for each of the selection traits were obtained with an inter-temporal farm model representing the production and economic circumstances of an average New Zealand dairy farm in 3 consecutive years from 1994 to 1996 (Harris, 1998). The economic value for each trait represented the discounted net income per 4.5 t of pasture DM, and they were a function of revenues and costs. In those years, the payment for milk for the producers was NZ$2.84/kg of fat, NZ$5.90/kg of protein, and a penalty value of NZ$0.04 per L of milk volume. The negative economic value for live weight reflected the higher feed cost of metabolizable energy for maintenance of the cow compared with the beef revenue per kilogram of live weight from culled cows and surplus animals.

Selection responses for live weight and lactation yields of milk, fat, and protein were calculated from the regression of the trait values on the selection index, assuming that EBV were obtained from best linear prediction (Henderson, 1963). Estimates of genetic and phenotypic parameters used in the selection index were taken from Spelman and Garrick (1997). The standard deviation of T was calculated to be NZ$26. Reliabilities of genetic evaluations (the squared correlation between T and BW) were obtained based on the number of lactation records for individual cows and from information on the 60 to 85 first-crop progeny-tested daughters for bulls.

Selection of animals to be parents of the next generation was undertaken by truncation across age classes following Ducrocq and Quaas (1988). The effect of selection of cows to breed cows was considered to be negligible. Bull mothers were selected from those with at least 3 generations of artificial breeding ancestry, provided they had at least seven-eighths gene composition of one breed. For each young bull to be progeny tested, 6.6 cows were contract mated. Within breed, the selected active cows that became bull mothers were mated to the 3 best bulls, which were selected from 5-, 6- and 7-yr-old proven, dead or alive, bulls. Results of progeny tests were obtained when bulls were 5 yr old. Bulls to breed cows were selected from live 5-, 6-, and 7-yr-old bulls. Numbers of bulls selected depended on anticipated future national demand for semen.

The model predicted the breed composition of the national herd for each of 9 mating strategies involving Holstein-Friesian (**F**), Jersey (**J**), and Ayrshire (**A**) breeds. The mating strategies simulated were as follows: straightbreeding; upgrading to F; upgrading to J; upgrading to A; 2-breed rotational FJ, FA, or JA; 3-breed rotational FJA; or use of the best bulls based on aggregate genetic merit regardless of breed. In all mating strategies, no fewer than 44,000 straightbred cows of each breed were retained to maintain a source of straightbred bull mothers.

Correlated responses, effects of heterosis, and age adjustment factors were included in calculating realized industry phenotypic averages for live weight and yields of milk, fat, and protein per cow. Environmental factors, such as temperature and annual rainfall, and complex interactions between animal and pasture that contribute to herd-year effects were not included in the model. Accordingly, the predicted yields of milk and its components for the various breed-age groups did not exhibit annual fluctuations from these causes, and might therefore be different from the actual yields.

In 1996, the year considered to be the base year of the simulation, F was the dominant breed, comprising 60% of the total population (Figure 1a). The model predicted that, based on the 1996 breeding objective, upgrading to J in combination with selection appeared to be the best option to increase dairy farm productivity and profitability. However, in practice, the adoption of upgrading to J did not occur as predicted, influenced at least in part by the continuous world-market decrease in the value for fat that was reflected in ongoing annual updates to the payment system for milk. Furthermore, breeding companies started progeny-testing crossbred bulls, and many farmers opted for the use of the best bulls regardless of breed, as suggested in the simulation study (Lopez-Villalobos et al., 2000a). Figure 1 shows that under this mating strategy, the model predicted the national breed composition for 2018 to be 11% F, 34% J, 52% F×J, and 2% A, whereas the actual breed composition was 36% F, 9% J, 53% F×J and 1% A (LIC/DairyNZ, 2019). The realized proportions of A and F×J crossbred cows in the national herd were very close to the proportions predicted by the model. However, the realized proportions for F and J straightbred populations were almost opposite. This disparity between the predicted and actual proportion of the straightbred populations was likely due to the continuous decrease in the value for fat and the perceived advantage of F animals for beef production instead of using the best J bulls.

**Figure 1:**
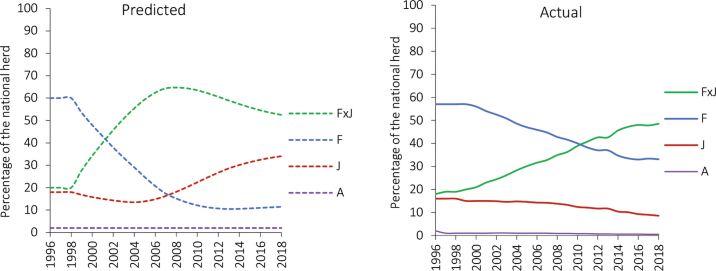
Predicted and actual (LIC/DairyNZ, 2019) breed composition of the New Zealand dairy herd from 1996 to 2018. F = Holstein-Friesian, J = Jersey, A = Ayrshire, and F×J = Holstein-Friesian × Jersey crossbreds.

Table 1 presents the actual phenotypic trends, the model predicted genetic trends and the actual genetic trends for milk traits and body weight. The actual genetic trends represent the retrospective breeding values of sires based on a single national evaluation from February 2020 (DairyNZ, 2020). The predicted and actual trends are very similar, indicating that the model was capable of accurately representing selection schemes within breed and use of bulls for purebreeding and crossbreeding in the commercial population. The model at that time did not consider the adoption of genomic selection, which started in 2008 (Spelman et al., 2013), but industry use of young bulls has still not exceeded that of proven sires. The actual genetic trends in the breeding values of sires in the last years do not demonstrate increased genetic gain for fat or protein after the implementation of genomic selection as observed in the US dairy cow population (García-Ruiz et al., 2016).

**Table 1:** Genetic trends in the New Zealand cow population predicted by a model 22 yr ago, actual genetic trends in sire breeding values with reliability >75%, and actual phenotypic trends for lactation yields of milk, fat, and protein and BW per cow

Trait	Actual phenotypic trend	Genetic trend predicted by the model	Actual trends in proven sires
Milk (L)	45.6	16.7	13.6
Fat (kg)	2.67	1.20	1.31
Protein (kg)	2.30	1.47	1.17
BW (kg)	−0.39	−0.72	−0.36

Fertility was not considered in the breeding objective or selection index because there has always been cow pathway selection to avoid impaired fertility as cows need to calve every 365 d on a pastoral farming enterprise. Fertility is now starting to get more emphasis with the increase in genomic evaluation of young bulls, but this is unlikely to have affected the national cow population at this stage.

The actual phenotypic trends and model-predicted and actual genetic trends for lactation yields of milk, fat, protein, and body weight per cow are shown in Figure 2. The actual phenotypic trends were greater than the actual- and model-predicted genetic trends, indicating that some of the production gains most likely resulted from improvements in management. In particular, New Zealand farmers transitioned from pasture-only management systems with virtually no supplementary feeding to systems that provided some supplementation of milking cows, particularly in the form of maize silage and palm kernel extract (Ledgard et al., 2020), which would account for much of the additional phenotypic gains in excess of the genetic gain.

**Figure 2:**
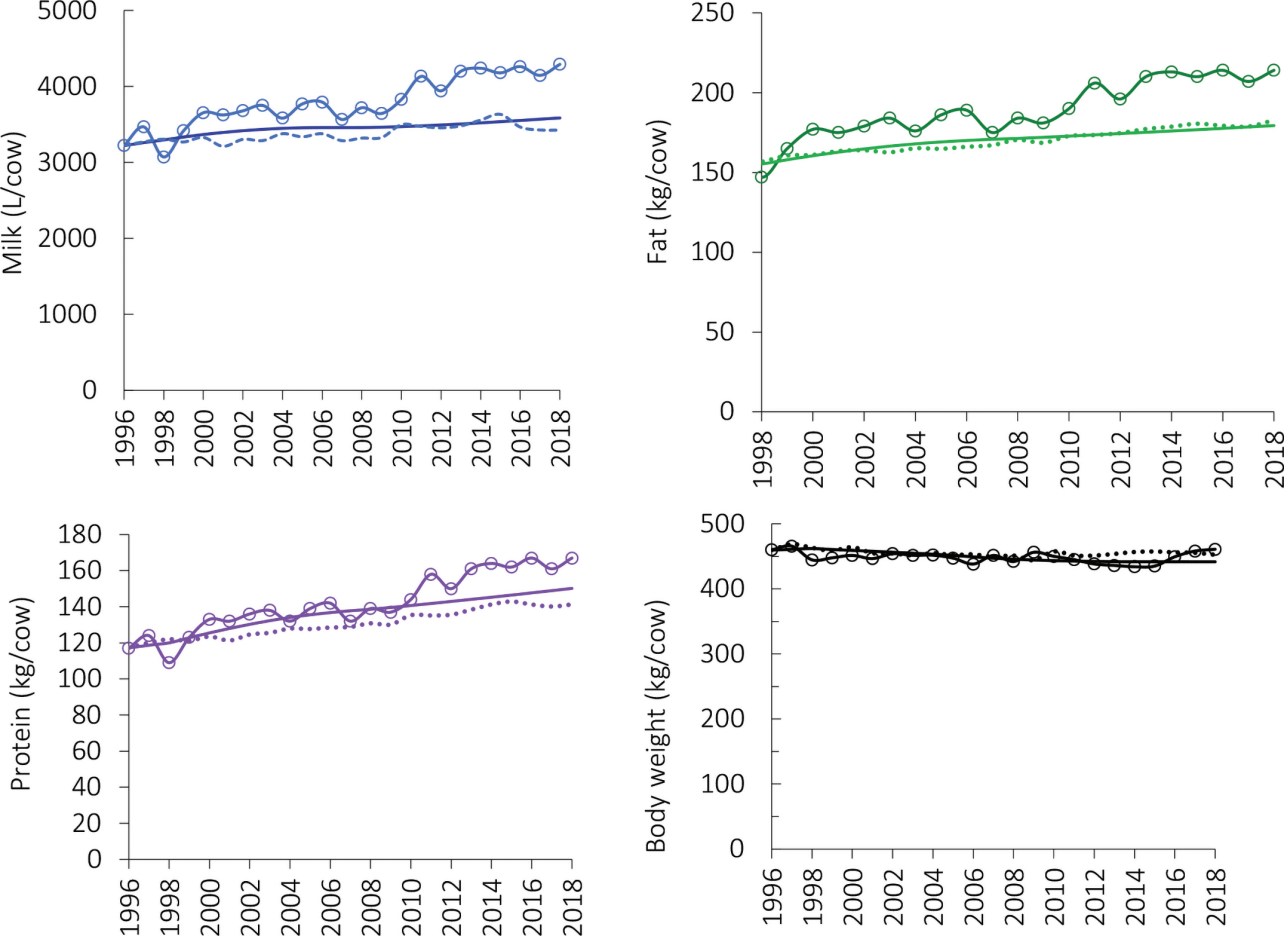
Actual phenotypic trends (LIC/DairyNZ, 2019; solid lines with open circles), model-predicted genetic trends (solid lines), and actual genetic trends in sire EBV (DairyNZ, 2020; dotted lines) for lactation yields of milk, fat, and protein, and BW of the average New Zealand dairy cattle population from 1996 to 2018.

Despite many national and international changes in political, economic, and management circumstances, the predicted long-term responses to selection and crossbreeding options closely mirrored predictions. This outcome should give confidence to scientists, decision-makers, and funders that long-term predictions of the genetic effect of technological developments can be accurately modeled.
