# Erratum to “Cumulative dairy cow genetic change from selection and crossbreeding over the last 2 decades in New Zealand closely aligns to model-based predictions published in 2000” (JDS Commun. 2:51–54)

**DOI:** 10.3168/jdsc.2022-3-2-164

**Published:** 2022-03-16

**Authors:** N. Lopez-Villalobos, H.T. Blair, D.J. Garrick

An animal ethics statement was missing from this paper. The following statement should be included: “No animals were used in this study, and ethical approval for the use of animals was thus deemed unnecessary.”
